# Rapid-Response and Wide-Range pH Sensors Enabled by Self-Assembled Functional PAni/PAA Layer on No-Core Fiber

**DOI:** 10.3390/ma15217449

**Published:** 2022-10-24

**Authors:** Gang Long, Liang Wan, Binyun Xia, Chao Zhao, Kunpeng Niu, Jianguo Hou, Dajuan Lyu, Litong Li, Fangdong Zhu, Ning Wang

**Affiliations:** 1 National Engineering Research Center of Fiber Optic Sensing Technology and Networks, Wuhan University of Technology, Wuhan 430070, China; 2 State Key Laboratory of Optical Fiber and Cable Manufacture Technology, Yangtze Optical Fibre and Cable Joint Stock Limited Company, Wuhan 430073, China; 3 Ningbo Lianghe Road & Bridge Technology Co., Ltd., Ningbo 315201, China

**Keywords:** no-core fiber, polyaniline, polyacrylic acid, pH sensor, multimode interference

## Abstract

The measurement of pH has received great attention in diverse fields, such as clinical diagnostics, environmental protection, and food safety. Optical fiber sensors are widely used for pH sensing because of their great advantages. In this work, an optical fiber pH sensor is fabricated, by combining the merits of the multimode interference configuration and pH-sensitive polyaniline/polyacrylic acid (PAni/PAA) coatings, which was successfully in situ deposited on the no-core fiber (NCF) by the layer-by-layer (LBL) self-assembly method. The sensors’ performance was experimentally characterized when used for pH detection. It has a high sensitivity of 0.985 nm/pH and a great linear response in a universal pH range of 2–12. The response time and recovery time is measured to be less than 10 s. In addition, its temperature sensitivity is tested to be about 0.01 nm/°C with a low temperature crosstalk effect, which makes it promising for detecting pH in the liquid phase with temperature variation. The sensors also demonstrated easy fabrication, good stability, and repeatability, which are adapted to pH detection in most practical applications.

## 1. Introduction

As known, pH-conditions regulation is of significance in the fields of biochemical industry [1], food safety [2], disease diagnosis [3], environmental engineering [4], and so on. A slight variation in pH conditions will cause the failure of crucial processes within the above domains [5]. Therefore, how to detect various environmental pH conditions accurately and quickly is essential for guiding their precise regulation. A variety of pH detection techniques have been developed, such as pH test paper [6], glass electrodes [7], biological [8], and quantum dots fluorescent [9]. Conventional pH test paper has been widely used, despite its low precision and large errors due to subjective visual judgment. Although glass electrodes have demonstrated high precision, they still show many shortcomings, such as low sensitivity, bulk size, insensitive response, etc. They also need to be recalibrated for each measurement, which limits their development toward online monitoring applications.

Optical fiber sensors have attracted considerable attention due to their small size [10], light weight [5], high sensitivity [11], remote operation for real-time sensing [12], etc., which have been studied and used widely for biochemical sensing applications. Among them, many optical fiber pH sensors have been extensively reported [12,13,14,15]. For example, Mishra et al. [16] proposed a fast-response, wide-range pH sensor by coating a smart hydrogel on a long-period fiber grating (LPFG) surface. Li et al. [17] coated the PAA/chitosan (CS)-sensitive film on the surface of the gold-coated optical fiber through the LBL self-assembly technique. The surface plasmon resonance (SPR) sensor realizes the high sensitivity detection of pH by utilizing a characteristic of the sensitive film: expanding/contracting with the change of pH. Yan et al. [18] reported a pH sensor with a Mach–Zehnder interferometer (MZI) cascaded with a fiber Bragg grating (FBG), by coating the graphene oxide/polyvinyl alcohol (GO/PVA)-sensitive film for pH detection, while the FBG was used for temperature calibration, which realized the temperature-self-calibrating pH monitoring. However, the disadvantages of complex optical fiber structures and fabrication processes have hindered their practical application. However, using some inexpensive polymer-sensitive materials combined with simple optical fiber architectures seems to be a promising pathway.

Various pH-sensitive materials have been used as coatings on the surface of optical fiber sensors to achieve highly sensitive detection of pH [19,20,21,22,23,24]. PAni is used for pH sensing because of its unique variable pH optical properties [25,26]. PAni was gradually deprotonated and converted from ES (emeraldine salt) to EB (emeraldine base) with increasing pH [27]. This results in a change in the doping and conformation of the polymer chain and, thus, a change in its refractive index (RI). PAni has excellent stability, physicochemical properties, and rapid and reversible adsorption or desorption kinetics. For the above reasons, PAni is widely used in various optical or electrical sensors. However, when PAni was deposited on the sensors’ surface using in situ oxidation polymerization, the PAni coating was completely not uniform for thicknesses lower than 2 µm [25]. When PAni powder was used, the coating was normally performed by the dip-coating method, and it tended to peel off. PAA is also an excellent pH-sensing material [28,29]. The polymer chain of PAA is rich in carboxyl groups. The degree of ionization of carboxyl groups varies with the changing of the pH condition. However, with the environmental pH increasing, PAA ionizes and expands into a fully solvated open-coil conformation, especially under a strong alkaline condition [29]. Therefore, PAni and PAA are unfavorable for pH sensing individually.

In this work, optical fiber pH sensors with a rapid response and a wide range are proposed by depositing the pH-sensitive polyaniline/polyacrylic acid (PAni/PAA) composite film on the fibers configured with multimode interference, composed of two pieces of single-mode fiber (SMF) and one section of no-core fiber (NCF). Here, benefiting from their differing electronegativity, the PAni/PAA composite coatings were in situ fabricated onto the NCF easily by using the typical layer-by-layer (LBL) self-assembly method. In addition, the combination of PAA and PAni makes up for the each other’s defects mentioned above, when used for developing the optical fiber pH sensors. It was experimentally demonstrated that the proposed pH sensors possess high sensitivity, rapid response, and low temperature crosstalk under universal pH conditions.

## 2. Experimental Section

### 2.1. Materials

Aniline (C_6_H_7_N, 99.5%), ammonium persulphate (APS, 98%), PAA (Mw = 100,000, 35 wt% solution in water), sodium hydroxide (NaOH, 96%), hydrochloric acid (HCl, 36%), sodium chloride (NaCl, 99.5%), N, and N–dimethylformamide (DMF, 99.5%) were all purchased from Sinopharm Chemical Reagent Co., Ltd., Shanghai, China. All the reagents were used without any further purification. The NCF (OD: 125 µm) and SMF (G.652) were purchased from Yangtze Optical Fiber and Cable Co., Ltd., Wuhan, China.

PAni powder was first synthesized by the typical chemical oxidative polymerization method [30,31]. During the synthesis processes, 10 mL of 0.4 M HCl solution was prepared and divided into two equal volumes. Subsequently, 0.2 g of aniline and 0.189 g of APS were, respectively, added to the two HCl solutions and thoroughly stirred to mix them uniformly. After that, the two solutions were mixed and placed in a refrigerator (BCD–196DK) at 0 °C for 30 min. Then, they were laid at about 4 °C overnight. Finally, the reaction solution was taken out and centrifuged. The precipitate was then dried in a vacuum oven (DZF–6050ABF) at 80 °C overnight, and the PAni powder was obtained.

When preparing the PAA precursor and the PAni precursor, 300 mg of PAA was first dissolved in 20 mL of deionized (DI) water to obtain the PAA solution with a 15 mg/mL concentration. In addition, 40 mg of the synthesized PAni powder was added into 4 mL of DMF solvent, while sonicated for 10 min and then vigorously stirred overnight. The PAni–DMF solution was then diluted with 16 mL DI water to 2 mg/mL.

### 2.2. Fabrication of the pH–Sensing Probe

**Figure 1:**
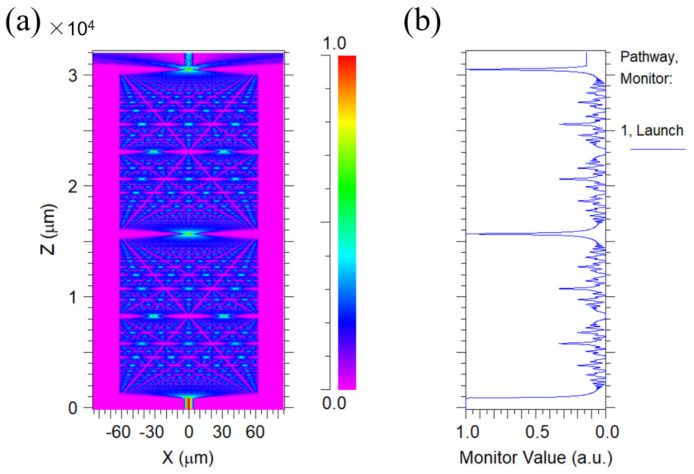
(**a**) Diagram of the optical field distribution inside a 3 cm NCF; (**b**) light–intensity values monitored on the Z–axis.

Before coating the PAni/PAA–sensitive film onto the NCF, the SNS (SMF–NCF–SMF) was fabricated first, and a section of NCF (length ~3 cm) was spliced with two SMF by a fusion splicer (FSM–100P, Fujikura, Shanghai, China). The NCF length was designed to be about 3 cm for the probe by considering the self–imaging effect of the NCF. To explain that more clearly, the simulation was conducted using the simulation software Rsoft 2013 BeamPRO, and the simplified mode was built. The dimensions of the simulated model are comparable to those of the actual sensor. The refractive index of the NCF is 1.463, and the length is 3 cm. The core refractive index of the SMFs is 1.4504, and the cladding refractive index is 1.4447. The background refractive index is set to 1. The incident light from the light source (λ = 1550 nm) was coupled into the NCF from a single–mode fiber that excites higher–order modes due to the mode–field mismatch effect, and the higher–order modes undergo multimode interference in the NCF. During the simulation, normalization was first conducted to make the maximum light intensity to be 1 at the center of the NCF. Figure 1a shows the internal optical field of the 3 cm NCF for the SNS probe. As the length of the NCF varies, the optical field is periodically in the present, which is the self–imaging effect. In addition, it was found that the light density reached the maximum when the light was transmitted to a point about 15 mm in the NCF. Thus, the self–imaging distance in the NCF could be regarded to be about 15 mm. If the length of the NCF is exactly equal or multiple to its self–imaging distance, the maximum output light would be obtained, which will be better for pH sensing. Figure 1b also shows the optical intensity distribution in the NCF. On the one hand, the longer the no–core fiber coated with sensitive materials is, the stronger the interaction between the light transmitted in the fiber and the material, which improves the sensitivity and accuracy of the sensing probe [32]. On the other hand, if the probe is too long, its actual application will be affected. Thus, the probe length was finally designed to be 3 cm.

**Figure 2:**
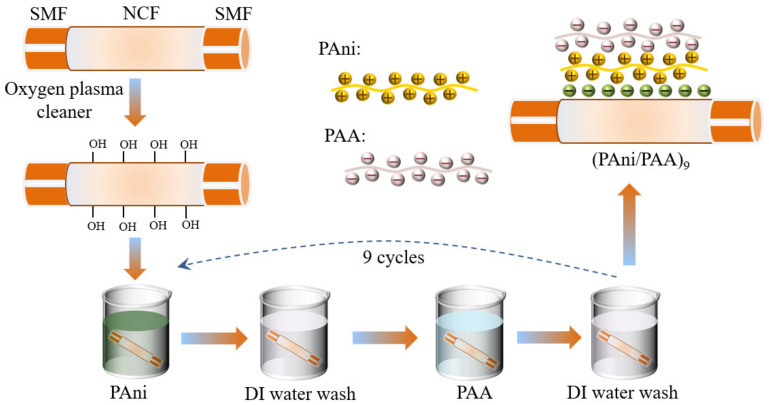
Fabrication of the SNS pH sensors by the LBL self–assembly method.

The PAni/PAA film was coated onto the NCF surface by the LBL self–assembly method, and the details are shown in Figure 2. The NCF section was treated using the oxygen plasma cleaner (power: 80 w; time: 300 s), which made the probe surface rich in hydroxyl groups and, thus, negatively charged. To ensure its smooth execution, the sensitive materials should be self–assembly deposited immediately after the plasma treatment, and the SNS probe was alternately immersed in cationic PAni–DMF solution and anionic PAA solution for 15 min, and the positive–charged PAni particles had the priority for naturally bonding with the hydroxylated glass fiber surface. The SNS probe was then rinsed with DI water for 3 min in between the immersions in cationic and anionic solutions, to remove the excess adsorbed components. The above steps were then repeated several times, and the self–assembled multilayers of PAni/PAA were obtained on the NCF. Finally, the coated SNS probe was delivered to the oven and dried at 60 °C overnight.

### 2.3. Characterization

The pH–sensitive films were characterized by scanning electron microscopy (SEM) (Zeiss Merlin Compact, Carl Zeiss, Germany, 15 kV resolution: 1.0 nm, 1 kV resolution: 1.7 nm, acceleration voltage: 1–20 kV, magnification: 20–300,000×). Fourier transform infrared spectroscopy (FTIR, wavenumber region: 4000–400 cm^−1^, number of scans: 32, resolution: 4 cm^−1^) spectra of the PAni, PAA, and PAni/PAA were recorded by the Thermo Scientific Nicolet 6700 spectrophotometer. The RI of different salt solutions was tested using an Abbe refractometer (WAY–2W, range of RI: 1.300–1.700, accuracy: 0.003).

### 2.4. Setup for pH Detection

**Figure 3:**
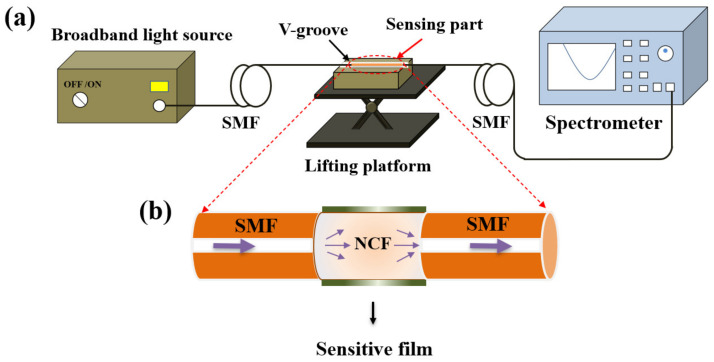
(**a**) Experimental setup for pH detection; (**b**) schematic diagram of the pH sensing probe.

Figure 3a shows the experimental setup for pH detection, which consists of the broadband light source (BBS, 1030–1660 nm), the V–groove, the lifting platform, the optical spectrum analyzer (OSA, YOKOGAWA AQ6370B, wavelength range: 600–1700 nm, high wavelength accuracy: ±0.01 nm, high wavelength resolution: 0.02 nm), and the SNS–sensing probe. The NCF coated with the pH–sensitive composite films were immersed in the V–groove filled with solution samples or rinsing regents, while connecting with the light source and the spectrometer by two sections of SMFs, which were fixed on both sides of the V–groove by rubber clamps to obtain the stable output of optical signals. The V–groove mounted on the lifting platform was employed to flexibly insert or remove the probe from the solution samples.

In the experiment, NaCl solutions with the same pH and different RI were prepared with RI of 1.3316, 1.3405, 1.3496, 1.3588, 1.3682, and 1.3773, respectively. The RI sensitivity of the bare SNS sensor was measured by immersing the sensing probe in NaCl solutions with different RI. To examine the pH detection performance of the sensor, the standard pH solutions were prepared by HCl or NaOH in DI water, with pH values from 2 to 12, and were calibrated by a commercial pH meter (HANNA, HI98103, range: 0.0 to 14.0 pH, resolution: 0.1 pH, accuracy: ±0.2 pH). To ensure data reliability, the sensor probe was immersed into the solution samples for 10 min, and the transmission spectrums were continuously monitored and recorded for each sample. Moreover, the response time, stability, and repeatability of this pH sensor were tested. To avoid the influence of temperature fluctuations on the measurement results, the temperature of the solution samples was kept at a constant room temperature (~25 °C). Afterwards, the influence of temperature on the pH sensor was investigated. The pH of the solution is 7, while the temperature is adjusted from 25 °C to 55 °C with an increment interval of 5 °C.

Figure 3b exhibits the schematic diagram of the sensor probe in detail. The NCF with a diameter of 125 µm was fused with two sections of SMFs, and the dark green layer representing the pH–sensitive composite films was coated just outside the NCF surface. When used for RI sensing, the NCF acts as the core, and the surrounding medium acts as the cladding. Multiple modes will be excited when the light enters the NCF from the input SMF, resulting in multimode interference. The interference spectrum dip wavelength of the mth order can be expressed:

(1) $$\lambda_{m}=\frac{2\Delta n_{eff}L}{2m+1}$$

where $\Delta n_{eff}$ is the effective RI difference between the fundamental and higher–order modes, L is the length of the NCF.

The pH variations of the samples will cause the RI of the coated pH–sensitive film to change on the surface of the SNS probe. At this point, the effective RI difference between the higher–order mode and the fundamental mode will change. This will eventually lead to a drift in the interference spectrum. The increase in RI of the PAni/PAA composite film reflects the increase in pH, which results in a red shift of the resonance wavelength. The interaction mechanism between the PAni/PAA composite film and the pH solution samples and their effect on the penetration in the SNS probe will be described in the next section.

## 3. Results

### 3.1. Characterization of the Sensing Probe

**Figure 4:**
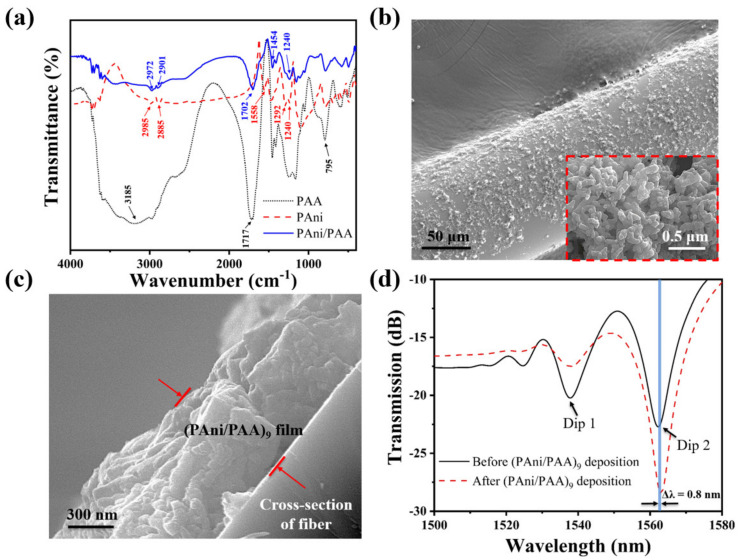
(**a**) FTIR spectra of PAni, PAA, and PAni/PAA; (**b**) SEM photos of the NCF with the PAni/PAA coatings; the inset is the scale–up image; (**c**) the cross–section of the NCF with the PAni/PAA coatings; (**d**) transmission spectrum of the SNS before and after the (PAni/PAA)_9_ deposition.

To confirm the successful attachment of the self–assembled film on the fiber surface, the pH–sensitive film on the fiber surface was characterized by FTIR. For comparison, the FTIR spectra of pure PAni and pure PAA were also measured. As shown in Figure 4a, the characteristic peaks of PAni present near the wavenumbers of 1558 cm^−1^ are ascribed to the stretching of the quinoid. In addition, the peaks around 1292 and 1240 cm^−1^ come from the aromatic C–N and C–N^+^ stretching vibration [33]. This demonstrates the successful synthesis of PAni. Pure PAA presented stretching–vibration bands of the carbonyl groups at 1717 cm^−1^ [34]. The peak of the PAA carbonyl group in the self–assembled film was centered at 1702 cm^−1^. Compared with pure PAA, the frequencies of C=O stretching in the self–assembled film were red–shifted by 15 cm^−1^. This indicates that strong intermolecular hydrogen bonds were formed between PAni and PAA during the self–assembly process [35]. The characteristic peaks of multilayers were present near the wavenumbers of 1454 cm^−1^ (C–C stretching mode for the quinoid ring) and 1240 cm^−1^ (C–N^+^ stretching vibration) [33,36,37]. This confirms the presence of the PAni in the coating. These results indicated that PAni and PAA were successfully attached to the fiber surface by self–assembly.

Figure 4b shows the scaled–up SEM image of the NCF coated with (PAni/PAA)_9_. Its original smooth surface becomes rough with noncontinuous coatings, which were composed of many nanoparticles observed from the scaled–up image in the Figure 4b inset, and it looks like a porous structure. Figure 4c exhibits the cross–sectional view of the sensitive coating on the NCF, and its thickness is about 1 µm. Figure 4d plotted the transmission spectrum of the SNS before and after (PAni/PAA)_9_ deposition in air. The shift of Dip 1 and Dip 2 are different after coating. Dip 1 did not shift, while Dip 2 red shifted by 0.8 nm from 1562.2 nm to 1563 nm. This is because the interference spectrum here is caused by multimode interference, and the response of each peak to the changing external environment reflectivity index is not consistent, which has also been reported and explained in detail [3,38].

### 3.2. RI Sensitivity of the Bare SNS Sensor

**Figure 5:**
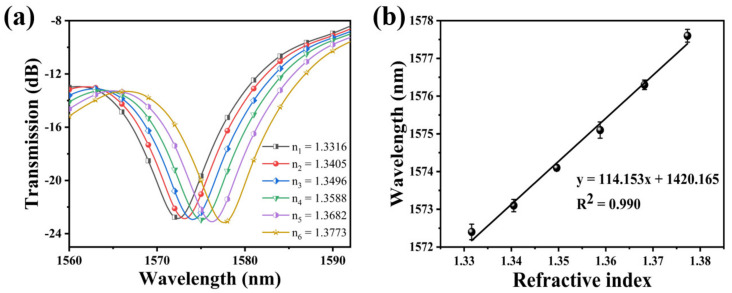
(**a**) Variation of the resonance wavelength of the bare SNS sensor with different RI; (**b**) the relationship between wavelength variation and the RI.

The transmission spectrum of the bare SNS is plotted in Figure 5a by immersing it in solutions with a different RI. It can be found that the resonance wavelength experiences a red shift when the RI increased. The relationship between the resonance wavelength and the RI exhibits a great linear fitting, and the RI sensitivity of this SNS sensor is 114.153 nm/RIU. The linear fitting is shown in Figure 5b. Its functional relationship is y = 114.153x + 1420.165 (R^2^ = 0.990).

### 3.3. pH Detection

**Figure 6:**
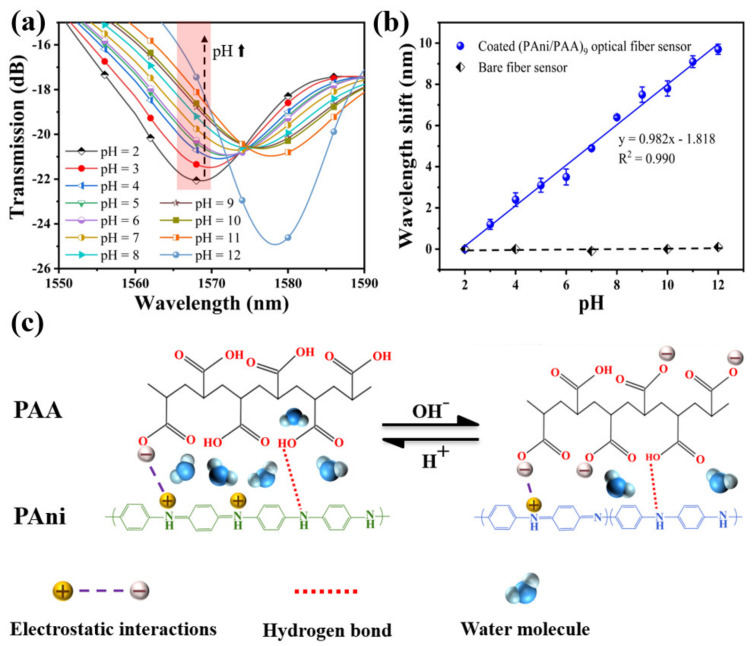
(**a**) Transmission spectra of the SNS sensor for different pH, ranging from 2 to 12 at 25 °C; (**b**) the fitting curve between resonant wavelength shift and pH; (**c**) schematic diagram shows the pH response of PAni/PAA sensitive film.

The transmission spectrums of the sensor in solution samples of different pH are shown in Figure 6a. When the pH value of the solution samples was adjusted from 2 to 12, the resonance peak shifted from 1568.6 nm to 1578.3 nm, and the total shift was about 9.7 nm. The relationship between the resonance wavelength and pH showed a great linear fitting of R^2^ = 0.990 in Figure 6b, and the function could be expressed by y = 0.982x − 1.818. Thus, its pH sensitivity can be defined as 0.982 nm/pH, in the pH range between 2 and 12. Here, the error bars are obtained by repeating the experiments five times. To further clarify the effect of the sensitive coating, a control experiment was also conducted, and the bare SNS without any coating was immersed into the solutions with different pH values (2, 4, 7, 10, and 12). There was no obvious variation for the resonant wavelength with the changing pH, indicating that the RI of the configured pH solutions is very close to that of the neutral DI water (1.3330). Therefore, the pH response of the PAni/PAA–coated SNS sensor is mainly caused by the RI change of the sensitive film induced by the different pH solutions.

The pH response characteristics of the sensor are consistent with the dissolution characteristics of the PAni/PAA–sensitive membranes for pH samples. As shown in Figure 6c, the imine (=N–) sites of PAni are protonated in an acidic medium during electrostatic self–assembly. The cation radicals (–NH_3_^+^) generated after PAni protonation are bound to the PAA network, containing anion radicals (–COO^−^) through strong electrostatic interactions and intermolecular hydrogen bonds, which results in a homogeneous sensitive film. With the increase in pH, the resonance wavelength underwent a red shift. This is because the thickness of the PAni/PAA coating changes with the pH. When the pH increased from 2 to 12, the PAA’s ionization degree continued to increase. That is, the number of –COO^−^ continued to increase. Although PAni gradually deprotonates and gradually changes from ES to EB, the overall degree of ionic cross–linking between the two still increases with increasing pH. This leads to the shrinkage of the multilayer membrane. As the thickness of the sensitive film decreases, the water molecules contained in the film gradually decrease, which causes the effective RI of the sensitive film to increase. This eventually leads to a red shift in the resonance wavelength.

### 3.4. Response Time

**Figure 7:**
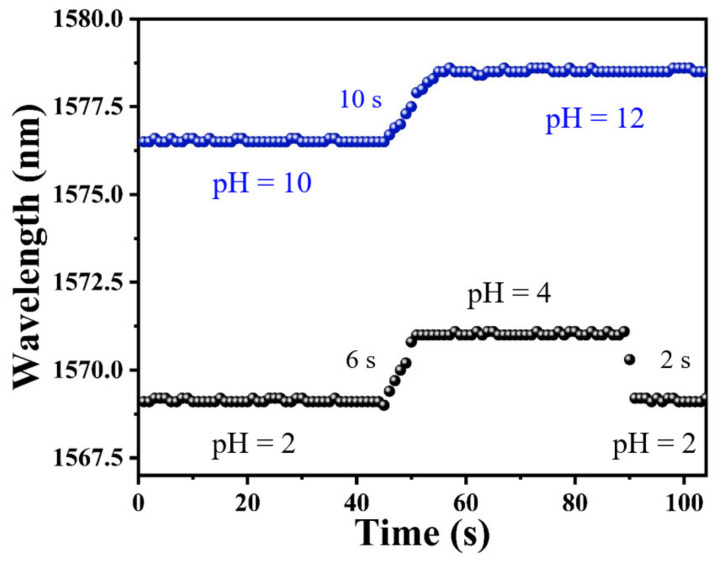
The response time of PAni/PAA–coated SNS sensor.

As one of the most important features of pH sensors, response time should be investigated to evaluate its performance for practical applications. The response time of a sensor is measured by a spectrometer. In addition, the dip wavelength temporal shifts were recorded by continuously scanning the transmission spectrum. During the experiment for testing the response time, the time resolution of the spectrometer for sampling was set as 1 s, and the data points in Figure 7 were recorded with an interval of 1 s. As shown in Figure 7, the pH response time from pH = 2 to pH = 4 was measured for a sensing probe coated with PAni/PAA–sensitive film. The sensor required only 6 s to achieve a stable spectral change from pH = 2 to pH = 4, and, correspondingly, the recovery time was 4 s faster than the response time. Under alkaline conditions, the response time was recorded to be about 10 s when the solution pH was adjusted from pH = 10 to pH = 12.

### 3.5. Stability and Repeatability of the pH Sensor

**Figure 8:**
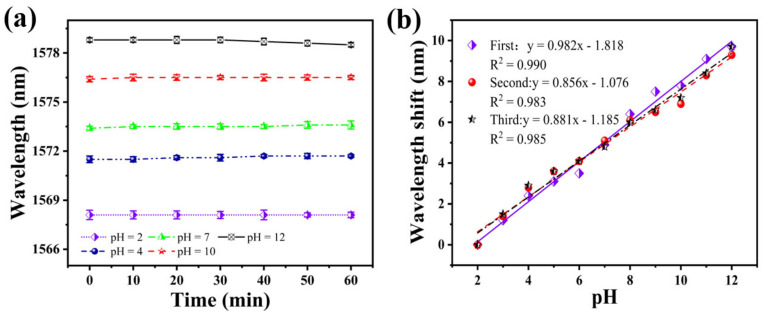
(**a**) Stability of the SNS sensor at different pH within 1 h; (**b**) shift of the resonance wavelength three pH tests on the same sensor, in the order of increasing pH.

To test the stability of the sensor, it was immersed in solutions with a different pH for 1 h. Figure 8a shows the fluctuation of the sensor spectrum at a pH of 2, 4, 7, 10, and 12, at 10 min intervals for 1 h. The fluctuation of the resonance wavelength is 0.1 nm in 30 min, considering the sensitivity of the sensor is about 0.982 nm/pH, which results in a measurement error of ±0.102 pH. When the pH is 12, the maximum fluctuation of the resonance wavelength in 1 h is 0.3 nm. Thus, it can be considered that the sensor’s stability curve remains flat within 1 h. Under other pH conditions, the fluctuation of the resonance wavelength is only 0.2 nm. This shows that the sensor has good stability.

Subsequently, to examine the repeatability of this sensor, we performed three pH tests in ascending order, using the same sensor. The results of the three repeated tests are shown in Figure 8b. The fitting curves of the three test results have a good coincidence. The average sensitivities were 0.982, 0.856, and 0.881 nm/pH, respectively. The similarity of the response characteristics and measurements’ detection sensitivity confirms the same sensor’s repeatability.

### 3.6. Effect of the Temperature

**Figure 9:**
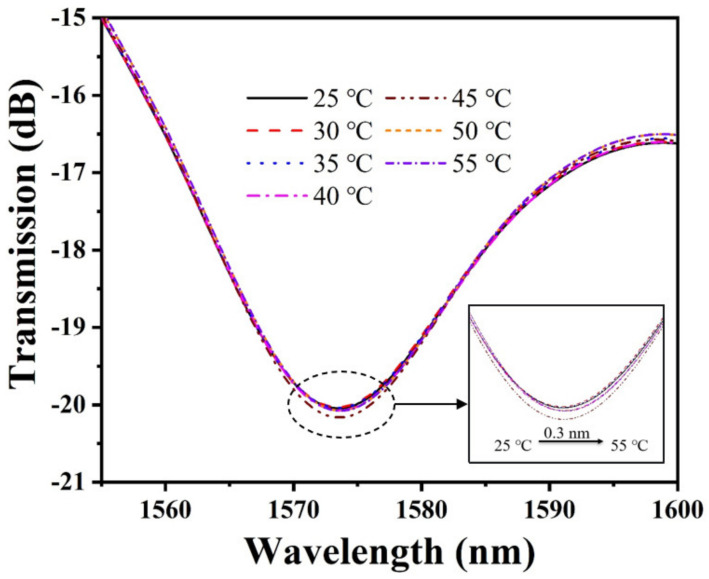
Temperature response of the pH sensor.

Many polymer–based pH sensors are subject to temperature crosstalk, considering that the ambient temperature may affect the pH of the solution and the physicochemical properties of PAni/PAA. Therefore, we investigated the response of temperature on the pH sensor. The experimental results are plotted in Figure 9. It can be noticed that the transmission spectrum shows a slight red shift with the increase in temperature. The main reason for this phenomenon is the gradual increase in the pH of the solution with the increase in the temperature. The increase in pH will cause the ionic cross–linking between PAni and PAA to increase, causing the shrinkage of the sensitive film. This causes an increase in the RI of the pH–sensitive film, which eventually causes a red shift in the interference spectrum. By calculation, the temperature sensitivity of the proposed pH sensor is only 0.01 nm/°C. At this point, the temperature cross–sensitivity of the sensor is approximately 0.0102 pH/°C. The temperature sensitivity of the proposed sensor is 1–2 orders of magnitude lower than the pH sensitivity, and its temperature crosstalk is lower than that of the previously reported optical fiber pH sensors. It is still applicable to some measurements without significant temperature variations.

## 4. Discussion

Here, the proposed sensors have been compared with other optical fiber pH sensors reported in previous studies, as shown in Table 1. The SNS pH sensor has a wide pH test range (pH~2–12), which meets the needs of most test environments, and has an excellent linear response to pH variation. The minimum response time of this sensor is only 2 s, which is better than those obtained by most pH sensors. Although the pH sensitivity of the PAA/CS based sensor [17] is larger, it also has higher temperature crosstalk and a lesser pH range. The sensor has excellent stability over a pH range of 3.18–11.84, and the measurement accuracy of about 0.16 pH is affected. Although the hydrogel-based LPFG sensor [16] has a shorter response time, its pH sensitivity is low, and its temperature cross-sensitivity is high. Obviously, it does not accurately detect the pH of the liquid environment. The GO/PVA-based sensor [18] cascaded the MZI and FBG to form a temperature self-calibrating pH sensor with excellent stability and good repeatability. Although this reduces temperature crosstalk during testing, it cannot ensure detection for universal pH conditions. The cascade complicates the sensor fabrication, which also limits its practical application. The PAni-based tilted fiber Bragg grating (TFBG) sensor [25] is adapted to wide-range pH measurements with a rapid response. However, the sensor has very low sensitivity, long stabilization times, and a large hysteresis. The pH-measurement range of the PAni-based tapered fiber sensor [26] is very narrow, and the sensitivity is low. The PVA/PAA-based photonic crystal fiber interferometer (PCFI) sensor [39] has a good repeatability but a narrow pH-measurement range. In other words, the sensor proposed in this work achieves a linear response, a fast response, and low temperature crosstalk in universal pH conditions, which obtains the trade-off performance when used for pH measurement.

## 5. Conclusions

In conclusion, this paper proposes a PAni/PAA-coated SNS optical fiber pH sensor, which was experimentally demonstrated with excellent trade-off performance when used for pH detection. To improve the sensors’ performance trade-off, PAni was synthesized by oxidative polymerization and in situ deposited a uniform PAni/PAA film on the surface of NCF by the self-assembly method, which successfully avoided their exfoliation and ensured the sensors’ stability and reliability. The experimental results show that the self-assembled membrane-based pH sensor has a good linear response in a universal pH range from 2 to 12, with R^2^ of 0.991 and large pH sensitivity of 0.985 nm/pH. The sensor’s response time and recovery time are less than 10 s. In addition, the proposed sensor has also been proven to have good repeatability and stability. Compared with other optical fiber sensors, the SNS sensor based on multimode interference avoids complicated fabrication processes, complex optical fiber structures, and delicate sensitive materials. Moreover, the simple fabrication and excellent performance make it attractive for pH measurement in various application fields.

## Figures and Tables

**Table 1:** Comparison of different optical fiber pH sensors.

Technique	pH Range	pH Sensitivity (nm/pH)	Minimum Response Time (s)	Temperature Sensitivity (nm/°C)	Stability	Repeatability
LPFG and hydrogel [16]	2–12	0.66	Less than 2	0.8	/	/
SPR and PAA/CS [17]	3.18–11.84	32.31	/	0.463	excellent	excellent
MZI cascading FBG and GO/PVA [18]	4–9.85	0.69	6	0.15 (MZI) 0.009 (FBG)	excellent	good
TFBG and PAni [25]	2–12	0.082	8	/	bad	/
Tapered fiber and PAni [26]	4–7 7–12	−0.54 0.28	/	/	/	/
PCFI and PVA/PAA [39]	2.5–6.5	0.9	12	−0.021 (pH = 3.52) −0.041 (pH = 4.68) 0.012 (pH = 5.82)	/	good
SNS and PAni/PAA (present study)	2–12	0.985	2	0.01	good	good

## Data Availability

Not applicable.
